# State Commissioners in Lunacy

**Published:** 1895-06

**Authors:** Grosvenor R. Trowbridge

**Affiliations:** 1331 Main Street; Buffalo, N. Y.


### STATE COMMISSIONERS IN LUNACY.

By GROSVENOR R. TROWBRIDGE, M. D., Buffalo, N. Y.

A rather peculiar condition exists in the present laws regarding the appointment of the commissioners in lunacy in the State of New York. As is known to all familiar with the make-up of this board, it consists of three individuals,—a physician, who shall always be president, a lawyer and a layman. So far, so good. Let us now consider the restrictions put upon the Governor in appointing these men. Although the following is not an absolutely accurate quotation of the law, nevertheless it gives the essential points. The physician must be a man of good morals, reputation and must have been in actual practice for ten years, and have had some experience in the care and treatment of the insane. The legal member and the layman have no such restrictions governing their appointments.

Now, why is all this red tape loaded upon the medical member? The law is unreasonable and absurd. It is easily seen that, under a "machine" administration (the present one is an exception in this state), a man who had been in practice ten years and had one day's experience in an insane hospital could hold the position, while a man who had had nine years and a half experience in one of our state institutions would not be eligible. This is not right, and the sooner the law is revised the better it will be. Why should the law not require that the legal member should have been in practice of his profession for ten years? Why does it not require that the layman should have been in business for ten years, and, finally, what earthly benefit will ten years in the practice of medicine be to a man who holds the position of medical member of the commission? He certainly should have nothing to say in regard to the actual treatment of the insane of this state, for that part is in the hands of our superintendents and their assistants. New York is, without question, in the lead in regard to the condition, management and quality of her state institutions and, without doubt, the present commission in lunacy is a good and efficient body, but heaven protect the unfortunates of our state should we have a second Altgeld in the gubernatorial chair.

1331 Main Street.

---

The Cross of the Legion of Honor is to be conferred upon Professors Behring, of Halle, and Löffler, of Griefswald, in recognition of their services in serum therapy. This is largely due to the action of Dr. Roux, who refused decoration unless the two German professors were also recognized.— *Chicago Medical Recorder.*

